# Multimodal agility-based exercise training (MAT) versus strength and endurance training (SET) to improve multiple sclerosis-related fatigue and fatigability during inpatient rehabilitation: a randomized controlled pilot and feasibility study [ReFEx]

**DOI:** 10.1186/s12883-023-03436-8

**Published:** 2023-10-28

**Authors:** Florian Wolf, Jörn Nielsen, Jochen Saliger, Eva Hennecken, Philipp Kröber, Mareike Eschweiler, Ann-Kristin Folkerts, Hans Karbe, Philipp Zimmer

**Affiliations:** 1 Neurological Rehabilitation Center Godeshoehe GmbH, Bonn, Germany; 2 Division of Performance and Health, Institute for Sport and Sport Science, Technical University Dortmund, Dortmund, Germany; 3 Department of Medical Psychology | Neuropsychology and Gender Studies, Center for Neuropsychological Diagnostics and Intervention (CeNDI), Faculty of Medicine and University Hospital Cologne, University of Cologne, Cologne, Germany

**Keywords:** Agility, Exercise, Fatigue, Multiple sclerosis, Rehabilitation

## Abstract

**Background:**

Multimodal agility-based exercise training (MAT) is a group-based exercise training framework for persons with multiple sclerosis (pwMS) with a potential to impact fatigue and fatigability. In a mixed-methods design, this study evaluated the feasibility of implementing MAT in an inpatient rehabilitation setting and the feasibility of a randomized controlled trial (RCT) study protocol with ‘traditional’ strength and endurance training (SET) as an active control condition. Secondarily, preliminary outcome data was acquired.

**Methods:**

PwMS with low to moderate disability and self-reported fatigue were randomly allocated to either MAT or SET when starting inpatient rehabilitation (4–6 weeks). The MAT-participants exercised in a group following a MAT-manual (sessions were gym- (5x/week) and pool-based (3x/week)). SET-participants exercised individually 5x/week on a cycle ergometer, and 3x/week on strength training machines. Feasibility assessments focused on processes, resources, management, time, and scientific domains. Assessed clinical outcomes at admission and discharge included perceived fatigue, motor and cognitive fatigability, cognitive performance, motor function, and balance confidence. Perceived fatigue was reassessed 1, 4, and 12 weeks after discharge. Feasibility was determined regarding predetermined progression criteria.

**Results:**

Twenty-two participants were randomized. Both groups performed the minimum number of sessions (> 18), and retention was adequate (73–91%). SET-participants performed more sessions than MAT-participants (30.8 vs. 22.7) and stayed longer in the facility (34.2 vs. 31.6 days). Non-eligibility of admitted pwMS was high (74% non-eligible), mainly due to high EDSS and inability to attend pool-based sessions. Consequently, recruitment (1.8/month) was slower than the predetermined progression criterium. Baseline assessments took longer than required (only 50% completed within 3 days). Short-term fatigue reduction was similar for both groups. Motor fatigability also improved in both groups, whereas cognitive fatigability deteriorated. In MAT, average improvement in walking endurance (43.9 m) exceeded minimal important change values for individuals (> 26.9 m).

**Conclusions:**

Progressing to a definitive RCT necessitates adaptation of eligibility criteria. In the present design it will also be difficult to attain similar dosing of interventions. A multicenter RCT focused only on gym-based MAT might be another option to assess the effect of MAT. The primary outcome measure should be able to measure change in perceived fatigue more robustly.

**Trial registration:**

German Clinical Trials Register: DRKS00023943, date of registration: 23 September 2021.

**Supplementary Information:**

The online version contains supplementary material available at 10.1186/s12883-023-03436-8.

## Background

Multiple Sclerosis (MS) is the most common, non-traumatic, neurological disorder among middle aged adults. Initially characterized as an inflammatory demyelinating disease of the central nervous system, neurodegenerative processes lead to progressive disability during later stages [1]. In Germany, persons with MS (pwMS) frequently attend inpatient rehabilitation facilities for several weeks to improve their ability to work in a multidisciplinary setting [2]. ‘Visible’ symptoms such as mobility impairments play an obvious role in pwMS’s ability to participate in the job market. However, 25% of pwMS are limited in their professional participation due to ‘invisible’ symptoms such as fatigue [3, 4].

The definition and conceptualization of fatigue has been changing and expanding for years, including recent updates [5, 6]. For the purpose of this study the term ‘fatigue’ refers to the ‘subjective sensation of lack of energy and exhaustion’ (p. E79) [7], retrospectively self-reported for a period of at least one week by a pwMS (i.e., the trait component of fatigue). The term ‘fatigability’ refers to objectively measured performance decrements on motor or cognitive tasks, corresponding to the taxonomy of Kluger et al. [8].

Contrary to its impact, pharmacological treatment options for fatigue are limited [9]. Consequently, many exercise and behavioral interventions have been evaluated [10]. One of the results concerning exercise is that endurance exercise, although frequently investigated, seems to have only a small effect on fatigue [11, 12]. Interventions broadly focused on ‘balance’ are less prevalent, but potentially with a more pronounced effect [10, 11]. Among exercise studies that explicitly addressed fatigue, almost none were conducted in an inpatient rehabilitation setting [11], which is characterized by a multidisciplinary environment, including various diagnostic and therapeutic components such as exercise, occupational therapy, health education, or neuropsychological assessment and training. Additionally, interactions between treatments as well as flexibility in the treatment schedule are common [13, 14]. This leaves clinical practice with few results that could be applied directly to this setting.

We have recently described a group-based exercise training framework for pwMS (multimodal agility-based exercise training [MAT] [15]), which might comprise several aspects that have been proposed to be beneficial for fatigue reduction, e.g., (I) balance training for making ‘navigating the environment’ less effortful [11], (II) ‘coordination of eye, head, and whole-body movements’ to ‘reduce the cognitive load associated with conscious compensatory strategies in dynamic environments’ [16], and (III) ‘improvement of sensory integration with a subsequent reduction of the cognitive load associated with motor processing’ [17]. As the MAT approach also includes other aspects suitable for inpatient rehabilitation (i.e., group-based, applicable to other neurological conditions [18]) and is proposed to be beneficial for several symptoms, including fatigability [15], the ReFEx (Rehabilitation, Fatigue, and Exercise) project aims to compare MAT with a ‘traditional’ exercise approach, namely, strength and endurance training (SET) [19] during inpatient rehabilitation. Both, strength and endurance training can be considered standard elements in neurorehabilitation facilities in Germany (and for the current clinic) and are part of national-level MS exercise guidelines [20].

In a first step, the present feasibility study was conducted to determine whether all aspects of the trial were implementable in the clinical setting, and to inform the potential progression to a powered randomized controlled trial (RCT). Secondarily, preliminary clinical outcome data was acquired.

## Methods

### Design and setting

The study was located at the Neurological Rehabilitation Center (NRC) Godeshoehe GmbH in Bonn, Germany, which provides neurorehabilitation for all levels of disability. It is certified by the German MS Society as one of three MS rehabilitation centers in the state of North Rhine-Westphalia and treats around 120 pwMS each year, 75% coming from within-state. All study-related sessions were implemented within existing therapy services of the NRC.

**Fig. 1:**
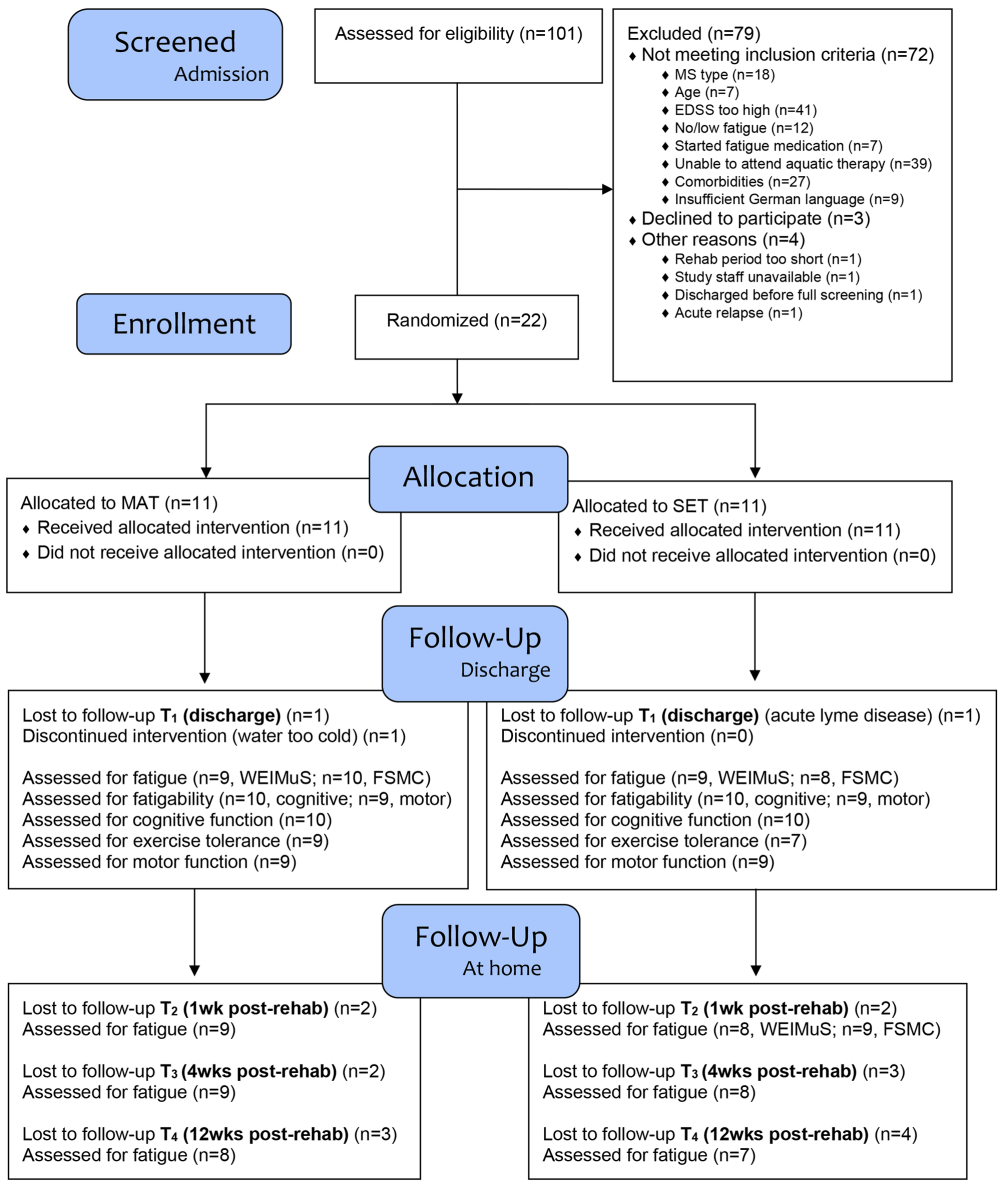
CONSORT diagram. EDSS=Expanded Disability Status Scale; FSMC=Fatigue Scale for Motor and Cognitive Functions; MAT=multimodal agility-based exercise training; MS=multiple sclerosis; SET=strength and endurance training; WEIMuS= Würzburg Fatigue Inventory for Multiple Sclerosis

The study had a two-armed, parallel-group, randomized-controlled design with 12 weeks follow-up (Fig. 1), pursuing a mixed-methods approach. The qualitative part will be reported elsewhere. We intended to recruit 12 participants per group [14], but no sample size calculation was performed as the feasibility evaluation was the primary aim.

Ethical approval was obtained from the Ethics Committee of the University of Bonn (reference number: 543/20). The study was prospectively registered in the German Clinical Trials Register (ID: DRKS00023943) on 23rd September 2021. For more details we refer to the published feasibility protocol [14].

### Screening and recruitment

New admissions were screened for MS and eligibility criteria were evaluated in a joint effort by the treating neuropsychologists (JN, JS, EH) and the principal investigator (FW). Inclusion criteria were a relapsing-remitting or secondary-progressive disease course (2017 McDonald criteria [21, 22]), age between 18 and 67 years (age for retirement in Germany), Expanded Disability Status Scale (EDSS) ≤ 5.0 [23], Fatigue Scale for Motor and Cognitive Functions (FSMC) ≥ 53 (cut-off for ‘moderate fatigue’) [24], and written informed consent. Exclusion criteria included the inability to attend aquatic therapy, comorbidities, that prevented attending study sessions, chronic neurologic conditions other than MS, insufficient German language skills, and specific fatigue medication (Amantadine, Modafinil) started less than 3 months ago. If deemed eligible, pwMS were informed about the study verbally and in written form.

### Randomization and blinding

After written informed consent, pwMS were randomly allocated (1:1) to MAT or SET, according to the minimization procedure [25], stratified by EDSS (≤ 3 or > 3), Würzburg Fatigue Inventory for Multiple Sclerosis (WEIMuS, < 38 or ≥ 38) [26], age (< 45 or ≥ 45), and MS disease course (relapsing-remitting or secondary-progressive). The WEIMuS acted as a stratification factor, as it was the potential primary outcome for a future RCT. Randomization was provided by an independent researcher from the German Sport University Cologne using RITA (‘Randomization-In-Treatment-Arms’, Evident, Germany).

The neuropsychological staff conducting the cognitive tests were blinded to the study groups. Participants, therapists, and staff conducting the physical tests and analyzing the questionnaires were not blinded regarding group allocation. However, participants were blinded regarding which of the groups was the experimental condition.

### Interventions

The intervention period lasted from admission (T_0_) to discharge (T_1_), comprising 4 to 6 weeks (based on medical indications, determined by the treating physician). MAT-participants performed five 30 min sessions of gym-based MAT, and three 30 min sessions of pool-based MAT per week in a group setting (including other neurological patients). SET comprised five 22 min sessions of endurance training on cycle ergometers, and three 30 min sessions of individual strength training. Endurance training was provided on cycle ergometers and not on a treadmill to enable more pronounced differences regarding the demand for sensory integration between MAT and SET [27]. Furthermore, it is the standard modality for endurance training in this clinic. Importantly, participants from both groups also attended a group on body awareness and relaxation techniques, which is part of usual care for pwMS in this clinic and which provided some social contact in the SET-group as well [14].

MAT consisted of three components: (I) standing balance exercises, (II) dynamic balance exercises including functional leg strength, and (III) agility-like exercises. Agility-like exercises have been defined as ‘[…] tasks, that require changes of direction, stop-and-go patterns, turns, and changing footwork strategies, with or without responding to a stimulus’ [15]. For load management in the gym-setting, three sessions with higher physical demands (i.e., agility-like components and functional leg strength) were interspersed with two sessions of lower physical strain (i.e., standing balance and exercises with a cognitive focus).

In SET, endurance training was performed with 3 min of gradual increase, 17 min steady and 2 min cool-down on a cycle ergometer (ergoselect 5, ergoline GmbH, Bitz, Germany) with continuous monitoring of power output (W) and heart rate (ers.2 software, ergoline GmbH, Bitz, Germany). The first session started with a power output participants had rated ‘light’ (= 11) to ‘somewhat hard’ (= 13) (6–20 Rating of Perceived Exertion [RPE] – scale) during the baseline graded exercise test (GXT, see Sect. 2.5.2 and Supplement) and then continued within this range. In detail, in each of the cycling sessions, therapists asked participants to rate their perceived exertion on the 6–20 RPE scale after 8 min and/or 15 min of cycling. Two timepoints were chosen in case perceived exertion changed during the session. If pwMS gave two different RPE ratings for each timepoint, the average score was documented. Therapists regulated the power output so that participants stayed between 11 and 13 on the RPE-scale. The new session always continued with the training load from the previous session. The range of 11–13 was chosen based on recent evidence-based recommendations for pwMS with similar EDSS [19].

Strength training was adapted from published protocols [17]: each session started with a 5 min warm-up, followed by three to four lower extremity exercises. Sessions 1 through 5 included 3 × 10 repetitions (intensity: 15 repetitions maximum [RM]) and Session 6 through discharge included 3 × 12 repetitions (12RM). More details and treatment manuals are displayed in the protocol [14].

### Outcomes

#### Feasibility

Feasibility domains were based on Thabane et al. [28] and evaluated according to prespecified progression criteria (Table 3). Evaluation of *processes* included the eligibility, recruitment, refusal, and retention rates, intervention adherence and fidelity. *Resources-*related outcomes focused on the number of days needed to complete baseline assessments, time requirements for the physical testing blocks at T_0_ and T_1_, and for the preparation of MAT sessions. *Data management* feasibility determined missing items from the WEIMuS and FSMC and missing assessments at T_0_ and T_1_. The *scientific* domain evaluated adverse events and perceived exertion. Adverse events were defined as events related to the interventions that led to early termination of a session. Perceived exertion was based on the session-RPE scale (0–10 scale, where 0 indicates ‘rest’, 10 indicates ‘maximum’ intensity) [29, 30]. See Table 4 in the protocol for details and rationales [14] and the Supplement for specifics of session-RPE application.

Six participants from each study arm were interviewed face-to-face at T_1_ regarding feasibility objectives (will be reported elsewhere).

#### Potential clinical outcomes

Perceived fatigue was assessed with the WEIMuS [26] and FSMC [24] questionnaires at T_0_ and T_1_. Participants were followed up via e-mail to fill out online versions 1, 4, and 12 weeks after discharge (T_2_-T_4_, Fig. 1). Change in WEIMuS total score from T_0_ to T_2_ was evaluated as the potential primary endpoint for a future RCT [14].

Other potential clinical outcomes for a definitive RCT were assessed only at admission and discharge and included cognitive fatigability (circadian changes in tonic alertness measures, assessed with the Test Battery of Attention Performance – Alertness [TAP-Alert] [31, 32] before 11 a.m. and after 3 p.m.), motor fatigability (Distance Walked Index [DWI] [33]), cognitive performance (California Verbal Learning Test [CVLT], Symbol Digit Modalities Test [SDMT] [34]), exercise tolerance (GXT on a cycle ergometer, protocol: start 25 W, progression 10 W/min, for details, see [35] and Supplement), motor function (6-Minute Walk Test [6MWT] [36], Timed 25-Foot Walk Test [T25FW] [37], Six Spot Step Test [SSST] [38], Functional Gait Assessment [FGA] [39]), and balance confidence (Activities-specific Balance Confidence scale [ABC] [40]).

### Data analysis

Descriptive statistics were used to summarize baseline sample characteristics, feasibility, and clinical outcomes, using IBM SPSS Statistics 29. Baseline differences between groups were examined using independent samples t-tests for normally distributed continuous variables or Mann-Whitney U tests for non-normally distributed and ordinal variables, and Fisher’s Exact test for categorical variables, with *p* < 0.05 indicating significant differences.

Change scores from baseline were calculated for clinical outcomes for each of the measurement timepoints, as was the frequency of participants in each group with a relevant improvement related to the WEIMuS (≤-6) [14] and FSMC (≤-10) [41] total scores at T_2_. Since this was a small-scale feasibility study, hypothesis testing of within- or between-group treatment effects was not performed [42, 43]. For the same reasons, no effect sizes were estimated [44]. However, we compared the feasibility data to the prespecified progression criteria (Table 3).

## Results

### Participants

Flow of participants is depicted in the CONSORT diagram (Fig. 1). Due to maintenance work of the pool starting in October 2022 we had to reduce the sample size from 24 to 22 participants. Baseline sociodemographic and clinical characteristics are described in Table 1.

**Table 1:** Baseline sociodemographic and clinical characteristics

	MAT (n = 11)	SET (n = 11)	*p*-value
**Age** mean (SD, min-max)	45.6 (10.1, 26–56)	53.3 (9.3, 31–64)	**0.019** ^a^
**Sex** f:m	10:1	8:3	0.586^b^
**BMI** mean (SD, min-max)	25.0 (5.0, 20.0–36.9)	28.9 (7.6, 21.6–48.0)	0.116^a^
**Work status** (n)	• Unfit for work (1) • Retired (3) • 3-6 h/d (3) • > 6 h/d (4)	• Unfit for work (1) • Retired (5) • 3-6 h/d (3) • > 6 h/d (2)	0.921^b^
**MS type** RR:SP	9:2	9:2	1.000^b^
**TSD** mean (SD, min-max)	9.5 (7.0, 2–28)	9.7 (8.6, 0–27)	0.699^a^
**EDSS** median (min-max)	3.0 (1.5–4.5)	2.5 (2.0–4.5)	0.748^a^
**Walking device** (n)	0	0	1.000^b^
**CES-D** mean (SD, min-max)	24.7 (11.7, 4–40)	25.7 (8.1, 13–39)	0.818^c^
**DMT** (n)	• None (4) • Glatiramer acetate (1) • Natalizumab (1) • Ofatumumab (1) • Teriflunomide (1) • Cladribine (1) • Fingolimod (1) • Siponimod (1)	• None (2) • Glatiramer acetate (2) • Natalizumab (1) • Ofatumumab (1) • Teriflunomide (2) • Dimethyl fumarate (1) • Interferone beta-1b (1) • Ocrelizumab (1)	0.929^b^
**Fatigue medication** (n)	• Amantadine (1)^*^ • None (10)	• None (11)	1.000^b^

^a^Mann-Whitney U test, ^b^Fisher’s Exact test, ^c^Independent samples t-test, bold = significant difference between groups. BMI = Body Mass Index; CES-D = Centre for Epidemiological Studies Depression Scale (German version); DMT = disease-modifying treatment; EDSS = Expanded Disability Status Scale; f = female; m = male; MAT = multimodal agility-based exercise training; max = maximum value; min = minimum value; MS = multiple sclerosis; n = number of patients; RR = relapsing-remitting multiple sclerosis; SD = standard deviation; SET = strength and endurance training; SP = secondary progressive multiple sclerosis; TSD = time since diagnosis in years

^*^this patient had started taking Amantadine more than three months ago and therefore, was not excluded

### Feasibility

Results regarding the *a priori* defined progression requirements are shown in Table 3.

#### Processes

Twenty-five of 101 (26%) patients screened were eligible and three of 25 (12%) declined to participate. Two were interested but overwhelmed with their current situation or wanted to focus on their own primary goals. One declined because not wanting to be restrained to one intervention. ‘EDSS’ and ‘able to attend aquatic therapy’ produced the most negative cases regarding eligibility, followed by comorbidities and disease course (Fig. 1, Table S1). It took 12 months (11/2021 to 11/2022) to randomize 22 participants, equaling 1.8 randomizations per month, which is below the progression requirement (Table 3).

Retention between T_0_ and T_1_ was 91% for each group. One MAT-participant dropped out during the intervention period, because of the pool being too cold. One SET-participant was excluded from the follow-up analysis as he developed acute lyme disease and was unable to attend most of the sessions and follow-up assessments. At T_2_, nine (82%, MAT-group) and eight (73%, SET-group) participants completed the WEIMuS, respectively. Thus, retention-related progression requirements were mostly fulfilled (Table 3).

Average length of stay in the rehabilitation facility were 31.6 (SD = 5.2, min-max = 25–41, n = 10) full days for the MAT-group and 34.2 (SD = 6.2, min-max = 22–41, n = 10) full days for the SET-group and both groups managed to attain the required minimum number of sessions (Table 3). However, on average, the SET-group performed more sessions than the MAT-group and adherence for the pool-based training was lower (76%) than for all other sessions (90–95%) (Table 2).

Regarding fidelity, a total of 122 gym-based and 76 pool-based MAT-sessions were logged and analyzed for MAT-components, as noted by the respective therapists. In an average week, 18.5%/17.9% (gym/pool) of training content targeted standing balance, 46.2%/50.1% dynamic balance/functional leg strength, and 35.3%/30.2% targeted agility-like exercises, showing that therapists provided all three MAT components. Average heart rate during all tracked gym-based sessions was 93.7 bpm (SD = 11.3, min-max = 78.8-114.1, n = 11), average maximum heart rate was 116.9 bpm (SD = 11.8, min-max = 99.1-132.3, n = 11).

In SET, strength sessions included an average 2.8 exercises (goal: three), and participants performed an average 99.5% of the prescribed load. Endurance sessions lasted for an average 21min26s (goal: 22 min) and average heart rate corresponded to 107.1 bpm (SD = 14.4, min-max = 90.5-127.9, n = 10). The average prescribed training intensity for cycling sessions was 57.5 W (SD = 24.2, min-max = 35–120, n = 10), while average actual load was 53.5 W (SD = 34.2, min-max = 24.5-139.6, n = 10), corresponding to an average 88% completion of the prescribed load (SD = 20, min-max = 54–116, n = 10). Average RPE (6-20) during cycling was 12.8 (SD = 0.5, min-max = 11.7–13.2, n = 10) (goal: 11–13).

**Table 2:** Adherence results are shown for MAT, SET and both groups combined (‘total’). Results are also given separately for gym/pool sessions and strength/endurance sessions

	Total n = 20	MAT n = 10	Gym n = 10	Pool n = 10	SET n = 10	Strength n = 10	Endurance n = 10
No. appointments (completed/scheduled (rate attended))	535/596 (90%)	228/270 (84%)	148/165 (90%)	80/105 (76%)	308/327 (94%)	117/123 (95%)	191/204 (94%)
Average completed sessions/participant (min-max)	26.8 (12–38)	22.7 (12–33)	14.8 (9–20)	8.0 (3–14)	30.8 (18–38)	11.7 (8–14)	19.1 (10–25)

MAT = multimodal agility-based exercise training; SET = strength and endurance training

#### Resources

Days needed to complete the baseline assessments were 4.1 (SD = 1.5, min-max = 3–9, n = 22) and 50% of participants completed all assessments within the first 3 days of therapy, which is below the required 80% (Table 3).

At T_0_ and T_1_, average time requirements for the physical assessments were 45 min (T_0_) and 43 min (T_1_) for motor function, and 30 min (T_0_) and 33 min (T_1_) for the GXT. To prepare MAT-sessions, therapists needed an average 3.7 min (gym-based, min-max = 1–12, n = 102 sessions) and 2.6 min (pool-based, min-max = 1–12, n = 62 sessions).

#### Data management

Data management revealed no missing items for WEIMuS and FSMC questionnaires and no missing assessments at T_0_. At T_1_, 7/22 (32%) participants had at least one missing assessment, with the GXT missing the most (six participants).

#### Scientific

No adverse events occurred in the MAT-group. During the cycling sessions, therapists noted six minor adverse events occurring in three participants (knee pain, severe fatigue, dizziness, low blood pressure).

Average session-RPE was 4.7 (gym-based, min-max = 2.3–6.8, n = 11, 141 sessions), 3.6 (pool-based, min-max = 2.3–5.4, n = 11, 61 sessions), 4.0 (strength, min-max = 1.7–5.4, n = 10, 105 sessions), and 3.8 (endurance, min-max = 2.1–5.8, n = 9, 136 sessions), respectively. According to the session-RPE scale a score of 3 indicates ‘moderate’ intensity, 4 is ‘somewhat hard’, and 5 is ‘hard’ [30].

**Table 3:** *A priori* progression requirements [14] and results

Requirement	Results
**quantitative**	
**1. Adherence**	
Average of at least 18 therapy sessions completed during the stay per group	+ MAT: 22.7 (12–33) + SET: 30.8 (18–38)
**2. Recruitment**	
4 participants/month < 25% non-eligible pwMS < 10% eligible but unwilling to participate	− 1.8/month − 74% non-eligible − 12%
**3. Retention**	
T_1_ > 90% per group T_2_ > 80% per group	+ 91% (both groups) + 82% (MAT) − 73% (SET)
**4. Time**	
> 80% able to complete all baseline assessments within the first 3 days of therapy	− 50%
**qualitative**	
**5. Interviews**	
Statements indicate that the interventions and study processes are acceptable	There were no major acceptability issues. Still, some adaptations to the study protocol were identified and will be reported separately.

Adherence data is presented as mean (min-max); + = requirement fulfilled; - = requirement not fulfilled; MAT = multimodal agility-based exercise training; pwMS = persons with multiple sclerosis; SET = strength and endurance training; T_1_ = discharge; T_2_ = one-week post-discharge

### Clinical outcomes

#### Fatigue

**Fig. 2:**
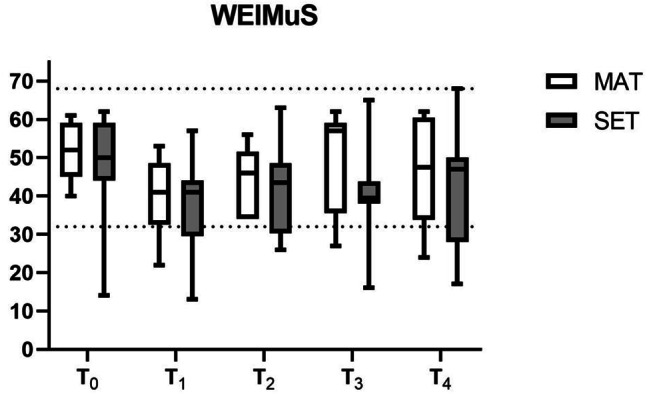
WEIMuS total scores for both groups. Lower scores indicate less fatigue. Box plot: line = median, whiskers = min-max. Upper dotted line = maximum WEIMuS total score (= 68); lower dotted line = cut-off for fatigue (= 32); MAT = multimodal agility-based exercise training; SET = strength and endurance training; T_0_ = admission; T_1_ = discharge; T_2_ = 1 week post-discharge; T_3_ = 4 weeks post-discharge; T_4_ = 12 weeks post-discharge

**Fig. 3:**
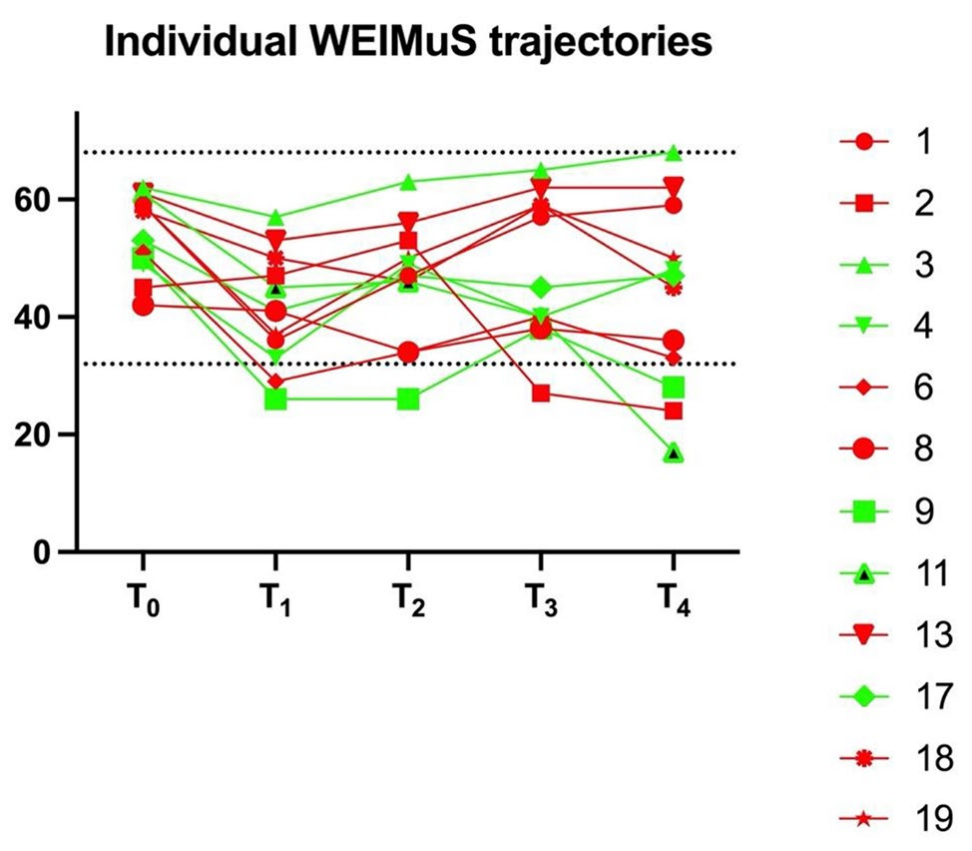
Individual WEIMuS total score trajectories for participants with a full data set from T_0_ to T_4_ (n = 12). A decrease in scores indicates less fatigue. Red = multimodal agility-based exercise training; green = strength and endurance training; upper dotted line = maximum WEIMuS total score (68); lower dotted line = indicates cut-off for fatigue [32]; T_0_ = admission; T_1_ = discharge; T_2_ = 1 week post-discharge; T_3_ = 4 weeks post-discharge; T_4_ = 12 weeks post-discharge

Both groups showed a comparable median reduction in WEIMuS total scores at T_1_ and T_2_ (Fig. 2, scores are reported in Table S2 as Supplement), with the MAT group having a higher percentage of participants with a relevant improvement at T_2_ (7/9, 78% [MAT] vs. 5/8, 63% [SET]). At T_3_, the MAT group displayed a sharp rise in scores, which dropped again at T_4_. From T_0_ to T_4_, individual WEIMuS trajectories showed an increasing variability for participants with a full data set (Fig. 3).

FSMC total scores are reported in the Supplement (Figure S1, Table S2). The proportion of participants with a relevant improvement at T_2_ was considerably lower than for the WEIMuS (0/9, 0% [MAT] vs. 3/9, 33% [SET]).

### Fatigability

Of the whole sample, three MAT- and two SET-participants revealed clinically relevant (i.e., at least − 10% decrease in meters walked between the first minute of the 6MWT and the last minute) [33] walking fatigability at T_0_ and T_1_, respectively. Both groups improved their DWI (i.e., less drop-off in meters walked between minute 1 and 6, Table 4) with several participants showing substantial improvements (e.g., + 14.6%). However, as most participants also walked further at T_1_, some also showed a worse DWI at T_1_.

Cognitively, tonic alertness reaction times increased in both groups between morning and afternoon assessments at T_0_ and T_1_, reflecting cognitive fatigability [45]. Unexpectedly, in both groups, afternoon alertness measures deteriorated between admission and discharge, as did the difference between morning and afternoon assessments. Only in the SET reaction times in the morning were faster at T_1_. Overall, variability was high.

### Other clinical outcomes

Peak power output in the GXT increased in both groups, with higher change scores in SET. Validity criteria [46] for cardiorespiratory fitness testing were mostly not attained, except for perceived exertion (Supplement). Therefore, we changed our terminology to ‘exercise tolerance’ [47].

All motor function measures also increased in both groups. For the 6MWT, improvements (43.9 m [MAT], 26.3 m [SET]) exceeded measurement error on group-level (≥11.1 m) and in the MAT also substantially exceeded minimal important change values for patients with mild disability (≥26.9 m) [48].

**Table 4:** Descriptive data on clinical outcomes at admission, discharge, and change scores (T_1_-T_0_).

Domain	Outcome	MAT		SET	
		Mean (SD)	Min-max	Mean (SD)	Min-max
**Fatigability**	**DWI (%)**				
	T_0_^a^ T_1_^a^ T_1_-T_0_ (change) ↑	-7.8 (6.5) -6.5 (5.2), n = 9 1.7 (8.5)	-17.6–0.0 -15.5–0.0 -9.8–14.6	-7.6 (5.8) -5.4 (5.3), n = 9 3.4 (4.3)	-20.0–3.3 -11.6–3.1 -2.0–9.6
	**TAP-Alertness (afternoon-morning difference, ms)**				
	T_0_^b^ T_1_^b^ T_1_-T_0_ (change) ↓	12.2 (60.2) 36.2 (136.3), n = 10 22.6 (130. 9)	-108–119 -97–364 -154–332	8.2 (79.7) 10.6 (50.7), n = 10 25.6 (56.2)	-62–240 -26–148 -19–167
**Cognitive performance**	**CVLT (n words)**				
	T_0_^c^ T_1_^c^ T_1_-T_0_ (change) ↑	56.9 (10.7) 53.0 (12.5), n = 10 -3.9 (7.5)	37–71 38–74 -18–3	53.0 (10.0) 53.7 (11.3), n = 10 0.8 (11.4)	28–65 36–70 -20–22
	**SDMT** **(n pairs)**				
	T_0_^d^ T_1_^d^ T_1_-T_0_ (change) ↑	49.7 (9.1) 52.9 (10.6), n = 10 1.2 (5.8)	30–62 36–67 -10–9	49.2 (9.3) 52.0 (8.8), n = 10 3.6 (6.1)	35–63 38–69 -5–13
**Exercise tolerance**	**GXT (W**_**peak**_ **)**				
	T_0_^c^ T_1_^c^ T_1_-T_0_ (change) ↑	106.8 (37.1) 101.7 (32.8), n = 9 2.2 (6.7)	55–175 65–165 -10–10	110.5 (42.7) 130.7 (51.6), n = 7 14.3 (16.1)	55–225 95–245 0–40
**Motor function & confidence**	**6MWT (m)**				
	T_0_^c^ T_1_^c^ T_1_-T_0_ (change) ↑	499.0 (90.1) 544.8 (101.9), n = 9 43.9 (30.0)	366–631 399–700 -25–77	511.5 (89.6) 537.1 (87.0), n = 9 26.3 (32.8)	315–648 365–660 -14–81
	**T25FW (s)**				
	T_0_^d^ T_1_^d^ T_1_-T_0_ (change) ↓	5.20 (0.99) 5.01 (0.79), n = 9 -0.10 (0.40)	4.05–7.10 3.95–6.5 -0.61–0.65	4.98 (0.98) 4.80 (0.55), n = 9 -0.30 (0.61)	3.90–7.05 4.35–6.10 -1.10–0.50
	**SSST (s)**				
	T_0_^d^ T_1_^d^ T_1_-T_0_ (change) ↓	8.19 (2.42) 7.55 (2.21), n = 9 -0.51 (1.12)	4.95–11.37 4.91–10.72 -2.45–1.52	7.48 (1.80) 6.58 (1.33), n = 9 -0.92 (1.07)	5.52–11.62 4.90–8.85 -2.77–0.43
	**FGA** **(total score)**				
	T_0_^c^ T_1_^c^ T_1_-T_0_ (change) ↑	23.0 (18.0–26.0)^*^ 24.0 (19.0–26.5), n = 9 2.0 (-1.5–4.5)	14–28 14–30 -4–6	24.0 (23.0–27.0)^*^ 25.0 (23.0–28.0), n = 9 2.0 (0.0–2.0)	12–28 15–29 -1–3
	**ABC (total score)**				
	T_0_^c^ T_1_^c^ T_1_-T_0_ (change) ↑	70.5 (21.3) 67.8 (19.4), n = 10 0.2 (7.2)	24.1–99.1 33.4–97.2 -12.5–10.3	75.8 (14.9) 72.1 (13.2), n = 8 -2.6 (6.8)	55.0–95.6 57.5–96.3 -16.4–5.6

All outcomes had n = 11 at T_0_, sample size for each outcome at T_1_ is reported in the table. All scores are presented as mean (SD, min-max), except for the FGA, presented as median (interquartile range). Arrows indicate direction for improvement. ^a^ = scores closer to zero indicate less fatigability; ^b^ = lower scores indicate smaller increase in fatigability from morning to afternoon; ^c^ = higher scores are favorable; ^d^ = lower scores are favorable; ^*^FGA scores are presented as median (interquartile range); 6MWT = 6-Minute Walk Test; ABC = Activities-specific Balance Confidence scale; CVLT = California Verbal Learning Test; DWI = distance walked index; FGA = Functional Gait Assessment; GXT = graded exercise test; MAT = multimodal agility-based exercise training; SDMT = Symbol Digit Modalities Test; SET = strength and endurance training; SSST = Six Spot Step Test; T25FW = Timed 25-Foot Walk Test; TAP = Test Battery of Attention Performance; T_0_ = admission; T_1_ = discharge; W_peak_ = peak power output

## Discussion

This study examined the feasibility of a trial comparing a ‘new’ group-based exercise framework for pwMS (MAT) [15] with ‘traditional’ exercise (SET) in an inpatient rehabilitation setting. Main clinical outcomes of interest for a definitive RCT were fatigue and fatigability [8].

Among the four predefined quantitative progression categories, one category was fulfilled (adherence), one was mixed, but approached a positive value (retention), and two were negative (recruitment, time). Therefore, changes to the study design are necessary.

Descriptively, favorable changes in fatigue were observable in both groups at the end of rehabilitation. Mobility related outcomes also improved in both groups, with the most pronounced changes in walking endurance, where MAT elicited clinically relevant changes.

### Feasibility

Advantages of inpatient rehabilitation include the high frequency of exercise sessions and high retentions rates, as patients are on-site. However, pwMS are also embedded in a multidisciplinary setting, with study-unrelated appointments possibly interfering with study-related sessions. Adherence rates were lower than the ones described by Zimmer et al. [49] (100%, n = 57) in a similar setting, but this trial also included fewer study-related sessions/week (3–5/week for 3 weeks vs. 8/week for > 4 weeks). Thus, the present results on adherence still indicate that a high frequency of sessions was possible on an organizational level and regarding the capacities of this (fatigued) patient collective. One exception was the pool session (only 76% adherence), which should be moved to a spot with a longer break from lunch, as indicated by pwMS in the interviews.

Another option would be to cut the pool-based sessions to (I) allow for more time to recover, (II) increase recruitment (as eligibility for pool-based sessions was low), (III) avoid the mix-up of effects from gym- and pool-based MAT, and (IV) lower the barrier for replication, without the need for a pool.

Even though both groups performed the minimum number of sessions, the SET-group attended substantially more sessions (228 [MAT] vs. 308 [SET]), as appointments were much more flexible due to individual scheduling. Consequently, MAT had considerably lower ‘dosing’. This is interesting, as one hypothesis of the MAT framework is that it might be more time-efficient [15]. Still, to evaluate differences in treatment effects it is important to ensure similar amounts of sessions performed, first [50].

Only about 1.8 participants were randomized per month instead of the intended four. There are examples of other trials conducted in similar settings, reporting equally low eligibility [49, 51]. If ‘able to attend aquatic therapy’ and ‘relapsing-remitting/secondary-progressive disease course’ would be excluded as criteria, eligibility could be increased from 26 to 35%. Still, this would not be sufficient. A preliminary sample size calculation for a clinically relevant difference regarding the WEIMuS retrieved a sample size of n = 66, which would take about 46 months to recruit with the present results, or about 32 months with the adapted inclusion criteria. A multicenter trial might be an option to progress in a more efficient amount of time. A limitation of the eligibility assessment could have been the COVID-19 pandemic, which might have resulted in fewer applications for rehabilitation from pwMS with lower EDSS, because of COVID-19 restrictions.

Interestingly, gym-based MAT elicited the highest ratings of session-based perceived exertion, which on average approached the rating of ‘hard’ - despite the fact, that average heart rate values were low. This warrants several considerations. First, the present heart rate values only give a very broad impression regarding intensity as they do not take maximum heart rate into account. As age differed between groups, heart rates indicating relative intensity would also be different. Furthermore, it is important to consider that even ‘high physical strain’ gym-based MAT sessions have many standing breaks, e.g., while the therapist gives demonstration and instructions, which profoundly reduces average heart rate for a complete session and leads to a more interval-like training stimulus. Values of average maximum heart rate might also be misleading, as ‘low physical strain’ MAT sessions blunted maximum values from the ‘high physical strain’ sessions. For example, four MAT-participants had individual gym-sessions with maximum heart rates above 150 bpm. Lastly, what we can only describe anecdotally is that MAT-participants repeatedly differentiated their session-based perceived exertion between physical and cognitive exertion (but were then prompted to give an overall score). Supposedly, this could reflect the unique content of MAT, and cognitive elements might increase session-RPE scores.

### Clinical outcomes

Regarding the performed assessments, this study is among the first which broadly focused on balance/motor control and conducted several follow-up measurements for fatigue [10]. Retention from end-of-treatment to 3 months follow-up was acceptable in both groups (78–89%), suggesting the online fatigue assessment to be a viable option for future follow-up assessments.

Observed (descriptive) changes in fatigue (i.e., WEIMuS) from admission to discharge/1 week after discharge were higher than for short-term treatment with fampridine, for example [52]. The reason for the rise in scores at T_3_ for MAT is unclear, but could be attributed to the small sample size, as scores dropped again at T_4_.

Proper assessment of fatigue seems to be one of the biggest challenges regarding the development of treatment strategies. The WEIMuS and FSMC differed in their classification of responders 1 week after discharge and clinically relevant change values have not been rigorously determined for these measures [7]. Therefore, switching to one of the ‘new generation’ questionnaires (e.g., PROMIS Fatigue (MS) 8a [53]) might be considered for a future trial [53, 54].

Similarly, no gold standard exists to quantify walking fatigability in pwMS [55]. Assessment of DWI is one option and was easily implementable in the present setting. Nevertheless, instrumented walking fatigability assessment has been advocated recently as another option and might be selected for a future RCT [55, 56].

Unexpectedly, cognitive fatigability tended to be worse at T_1_. A reason for this might be that the cognitive load between the morning/afternoon assessments was not rigorously standardized. In addition, it has been shown that a number of other disease-specific factors (e.g., daytime sleepiness, hand motor impairment), personality-related behavioral factors in dealing with performance situations (e.g., energy management, test anxiety), or psychometric aspects of test use (e.g., practice effects) could contribute directly or indirectly to this relationship, complicating interpretation [57].

### Limitations

As mentioned, advantages of inpatient rehabilitation are the high volume and frequency of exercise sessions and high retentions rates. However, some general disadvantages of the inpatient rehabilitation setting should be reconsidered. First, the intervention duration is restricted and, as MAT is a group-based, therapist-led intervention (i.e., no home-based or digital training) [15], the setting precludes the possibility of evaluating long-term MAT effects. If MAT is framed as a reserve-building activity long-term studies are needed [15, 58], which would favor an outpatient setting. Second, the multidisciplinary setting might affect some of the outcome measures. As it would be unethical to withhold a certain kind of therapy, patients can receive, for example, varying degrees of complementary computerized cognitive training, making it challenging to distill the effect of the study-related training. This feeds into the wider discussion on the ‘black box’ of usual care and treatment components in rehabilitation [59]. Third, the length of stay in a neurorehabilitation facility in Germany is not fixed but is determined during the stay by the treating physician based on medical indications and can vary between 4 and 6 weeks. Therefore, intervention duration was not matched. However, this allows the design to reflect the actual clinical setting and the same follow-up periods post-discharge. Lastly, all results from the present feasibility study, must be seen in light of the small sample size, and potentially ‘delivery agent bias’ [60], i.e., the newly evaluated intervention (MAT) was partially provided by a developer (FW) [14], which might have increased its effects and compromised blinding.

## Conclusions

Substantial changes to the study design are needed, especially to increase recruitment. Going forward, a multicenter trial focused on gym-based MAT might be another option. ‘New generation’ fatigue questionnaires, instrumented motor fatigability, and alertness assessments with a standardized cognitive load are candidates for improved outcome assessments.

### Electronic supplementary material

Below is the link to the electronic supplementary material.

Supplementary Material 1

## Data Availability

The raw data were generated at the Neurological Rehabilitation Center Godeshoehe GmbH. The datasets used and/or analysed during the current study are available from the corresponding author on reasonable request.

## List of Abbreviations

6MWT: 6-Minute Walk Test
ABC: Activities-specific Balance Confidence scale
CVLT: California Verbal Learning Test
DWI: Distance Walked Index
FGA: Functional Gait Assessment
FSMC: Fatigue Scale for Motor and Cognitive Functions
GXT: Graded exercise test
MAT: Multimodal agility-based exercise training
NRC: Neurological Rehabilitation Center
ReFEx: Rehabilitation, Fatigue, and Exercise
pwMS: Persons with multiple sclerosis
RPE: Rating Perceived Exertion
SET: Strength and endurance training
SDMT: Symbol Digit Modalities Test
SSST: Six Spot Step Test
T25FW: Timed 25-Foot Walk Test
TAP-Alert: Test Battery of Attention Performance – Alertness
WEIMuS: Würzburg Fatigue Inventory for Multiple Sclerosis
