# Supplementary material for: Multimodal agility-based exercise training (MAT) versus strength and endurance training (SET) to improve multiple sclerosis-related fatigue and fatigability during inpatient rehabilitation: a randomized controlled pilot and feasibility study [ReFEx]
Source: BMC Neurol. 2023 Oct 28;23:388. doi: 10.1186/s12883-023-03436-8 (PMC10612282; doi:10.1186/s12883-023-03436-8)
Supplement: Supplementary file 1 — Supplementary Material 1 [file 12883_2023_3436_MOESM1_ESM.docx]

**Multimodal agility-based exercise training (MAT) versus strength and endurance training (SET) to improve multiple sclerosis-related fatigue and fatigability during inpatient rehabilitation: a randomized controlled pilot and feasibility study [ReFEx].**

**Authors:** Florian Wolf, Jörn Nielsen, Jochen Saliger, Eva Hennecken, Philipp Kröber, Mareike Eschweiler, Ann-Kristin Folkerts, Hans Karbe, Philipp Zimmer

**Supplement**

**Detailed description of the graded exercise test (GXT)**

Due to the clinical setting, measuring cardiorespiratory fitness with the gold standard of spirometry was not possible for this pilot study. Nevertheless, we applied the formula described by Valet, Stoquart (1) to predict the peak oxygen consumption, using the peak power attained in the GXT on a cycle ergometer (ERGO-FIT Cycle 4000 med; ERGO-FIT GmbH & Co. KG, Pirmasens, Germany) (see Table S3). According to Valet, Stoquart (1) validity and reliability of this method was very good in pwMS (intraclass correlation coefficient = 0.75)(1). The protocol of the GXT was based on previous protocols, as currently, standard disease specific protocols do not exist(2). Specifically, it was based on a protocol, used for several other studies performed in a similar inpatient rehabilitation setting(2), with some slight differences due to the present participant sample: (a) workload always started at 25W, (b) it was increased by 10W/min for all participants until volitional exhaustion. The cool-down period consisted of 3min of light pedaling at 0-25W. Heart rate was continuously monitored using a Polar Verity Sense (Polar, Kempele, Finland) connected to an iPad (Apple, Cupertino, USA) with the Polar Team App. At the end of each minute, participants were asked to rate their perceived exertion using the 6-20 Rating of Perceived Exertion (RPE) – scale. Before starting the GXT, instructions regarding the RPE-scale were read to the participants according to Borg (3). Right after volitional exhaustion, participants were asked to pick a reason for test termination (Table S4).

**Detailed description of assessing the session-RPE**

The session-RPE gives a rating of perceived exertion for a complete session. Specifically, the modified Category Ratio (CR-10) RPE scale as developed by Foster, Florhaug (4) was used to quantify session-RPE.

Endurance training (cycling): Before the first session, participants received a ‘diary’, and were instructed to note their session-RPE right after finishing each of the cycling sessions. The diary also included: (I) the question participants were supposed to answer: “Overall, how strenuous was the cycling session today?”, (II) written instructions (“please do not rate how you feel overall today, or how much fun you had during cycling. Instead, only rate how strenuous the cycling was. There is no right or wrong. This is only about your personal impression”), (III) the session-RPE scale (in German). The diary form was chosen for the endurance training to reduce study-related obligations for the therapists.

All other sessions (strength, gym, pool): session-RPE was directly ascertained and documented by the respective therapist after finishing a session. Participants were specifically instructed to rate the complete session and not specific exercises.

**Table S1** Eligibility criteria, positive and negative cases.

| **Criteria** | **n/total** | **Percentage** |
| --- | --- | --- |
| **Disease course (RR/SP)** | | |
| Info missing | 1/101 | 1% |
| Eligible | 82/100 | 82% |
| Non-eligible | 18/100 | 18% |
| **Age (18-67)** | | |
| Info missing | 0/101 | 0% |
| Eligible | 94/101 | 93% |
| Non-eligible | 7/101 | 7% |
| **EDSS (up to 5.0)** | | |
| Info missing | 3/101 | 3% |
| Eligible | 57/98 | 58% |
| Non-eligible | 41/98 | 42% |
| **FSMC (53 and up)** | | |
| Info missing | 25/101 | 25% |
| Eligible | 41/76 | 84% |
| Non-eligible | 12/76 | 16% |
| **Fatigue medication (not started less than 3 months ago)** | | |
| Info missing | 1/101 | 1% |
| Eligible | 93/100 | 93% |
| Non-eligible | 7/100 | 7% |
| **Water therapy** | | |
| Info missing | 0/101 | 0% |
| Eligible | 62/101 | 61% |
| Non-eligible | 39/101 | 39% |
| **Comorbidities** | | |
| Info missing | 1/101 | 1% |
| Eligible | 73/100 | 73% |
| Non-eligible | 27/100 | 27% |
| **Language** | | |
| Info missing | 0/101 | 0% |
| Eligible | 92/101 | 91% |
| Non-eligible | 9/101 | 9% |

EDSS=Expanded Disability Status Scale; FSMC=Fatigue Scale for Motor and Cognitive Functions; RR=relapsing-remitting; SP=secondary-progressive.

**Table S2** WEIMuS and FSMC total scores.

| **WEIMuS** | | | | | |
| --- | --- | --- | --- | --- | --- |
|  | **T_0_** | **T_1_** | **T_2_** | **T_3_** | **T_4_** |
| **MAT**  Median  Change from T_0_ | n=11  52 (40 – 61) | n=9  41 (22 – 53)  -14 (-24 – 2) | n=9  46 (34 – 56)  -12 (-17 – 8) | n=9  57 (27 – 62)  -2 (-18 – 6) | n=8  47.5 (24 – 62)  -7.5 (-21 – 9) |
|  |  |  |  |  |  |
| **SET**  Median  Change from T_0_ | n=11  50 (14 – 62) | n=9  41 (13 – 57)  -12 (-30 – -1) | n=8  43.5 (26 – 63)  -13 (-15 – 1) | n=8  39.5 (16 – 65)  -10.5 (-27 – 3) | n=7  47 (17 – 68)  -5 (-44 – 6) |
| **FSMC** | | | | | |
| **MAT**  Median  Change from T_0_ | n=11  83 (61 – 93) | n=10  77.5 (74 – 89)  -4 (-18 – 6) | n=9  80 (74 – 91)  -3 (-8 – 4) | n=9  82 (68 – 92)  -4 (-16 – 9) | n=8  80 (68 – 98)  1 (-16 – 10) |
|  |  |  |  |  |  |
| **SET**  Median  Change from T_0_ | n=11  78 (65 – 100) | n=8  82 (65 – 91)  3.5 (-9 – 7) | n=9  72 (55 – 94)  -7 (-15 – 4) | n=8  78 (56 – 92)  -5.5 (-14 – 6) | n=7  75 (68 – 99)  -1 (-18 – 5) |

Values present median (min-max). Decrease in scores indicates less fatigue. FSMC=Fatigue Scale for Motor and Cognitive Functions; MAT=multimodal agility-based exercise training; SET=strength and endurance training; T_0_=admission; T_1_=discharge; T_2_=1 week post-discharge; T_3_=4 weeks post-discharge; T_4_=12 weeks post-discharge; WEIMuS=Würzburg Fatigue Inventory for Multiple Sclerosis;

**Table S3** Morning and afternoon data of the alertness assessment and supplementary data from the graded exercise test.

|  | **MAT** | **SET** |
| --- | --- | --- |
| **TAP-Alertness (ms)**  **Morning**  T_0_^a^  T_1_^a^  T_1_-T_0_ (change) ↓  **Afternoon**  T_0_^a^  T_1_^a^  T_1_-T_0_ (change) ↓ | 306.8 (97.2, 230 – 572)  365.8 (193.8, 247 – 872), n=10  53.9 (95.8, -13 – 300)  319.0 (109.3, 216 – 590)  402.0 (193.8, 247 – 872), n=10  76.5 (126.4, -69 – 341) | 299.7 (84.6, 194 – 463)  278.7 (46.4, 216 – 373), n=10  -23.0 (56.5, -143 – 32)  307.9 (113.6, 195 – 520)  289.3 (71.6, 198 – 453), n=10  2.6 (82.6, -155 – 158) |
| **W_peak/kg_**  T_0_^b^  T_1_^b^  T_1_-T_0_ (change) ↑ | 1.50 (0.57, 0.74 – 2.41)  1.45 (0.48, 0.83 – 2.22), n=9  0.04 (0.09, -0.14 – 0.16) | 1.39 (0.59, 0.70 – 2.76)  1.60 (0.71, 0.93 – 3.09), n=7  0.17 (0.18, -0.03 – 0.47) |
| **_p_VO_2peak_ (ml/min)**  T_0_^b^  T_1_^b^  T_1_-T_0_ (change) ↑ | 1422.0 (370.3, 875.1– 2040.4)  1369.3 (336.8, 971.3 – 1947.3), n=9  15.1 (68.0, -93.1 – 98.5) | 1496.8 (409.2, 1079.2 – 2572.0)  1718.0 (497.5, 1319.2 – 2742.9), n=7  129.0 (158.4, -31.6 – 394.1) |

All scores had n=11 at T_0_, sample size for each outcome at T_1_ is reported in the table. All scores are presented as mean (SD, min-max). Arrows indicate direction for improvement. ^a^=lower scores are favorable; ^b^=higher scores are favorable; MAT=multimodal agility-based exercise training; _p_VO_2peak_=predicted^#^ peak oxygen uptake; SET=strength and endurance training; T_0_=admission; T_1_=discharge; TAP=Test Battery of Attention Performance; W_peak/kg_=peak power output relative to bodyweight.

^#^ _p_VO_2peak_=9.39*W_peak_ + 7.70*weight (kg) – 5.88*age (years) + 136.7 ml/min, according to Valet, Stoquart (1).

**Results of GXT validity criteria(5)**

1. Heart rate at W_peak_ within 90% of predicted maximum heart rate (208 – 0.7*age)?

- T_0_ Yes=3/22 (13.6%)
- T_1_ Yes=2/16 (9.1%)

1. Rating of perceived exertion (RPE 6-20) at W_peak_ ≥17?

- T_0_ Yes=22/22 (100%), mean (SD): 19.18 (0.73)
- T_1_ Yes=16/16 (100%), mean (SD): 19.44 (0.52)

**Table S4** Reasons for termination of the graded exercise test.

| Reason | T_0_ | T_1_ |
| --- | --- | --- |
| Breathing  Leg weakness  Pain  Sensory issues  Dizziness | 3/22 (17.6%)  **17/22 (77.3%)**  1/22 (4.5%)  0/22 (0.0%)  1/22 (4.5%) | 2/16 (12.5%)  **14/16 (87.5%)**  0/16 (0.0%)  0/16 (0.0%)  0/16 (0.0%) |

Data are presented as n/total (percentage). T_0_=admission; T_1_=discharge.

**
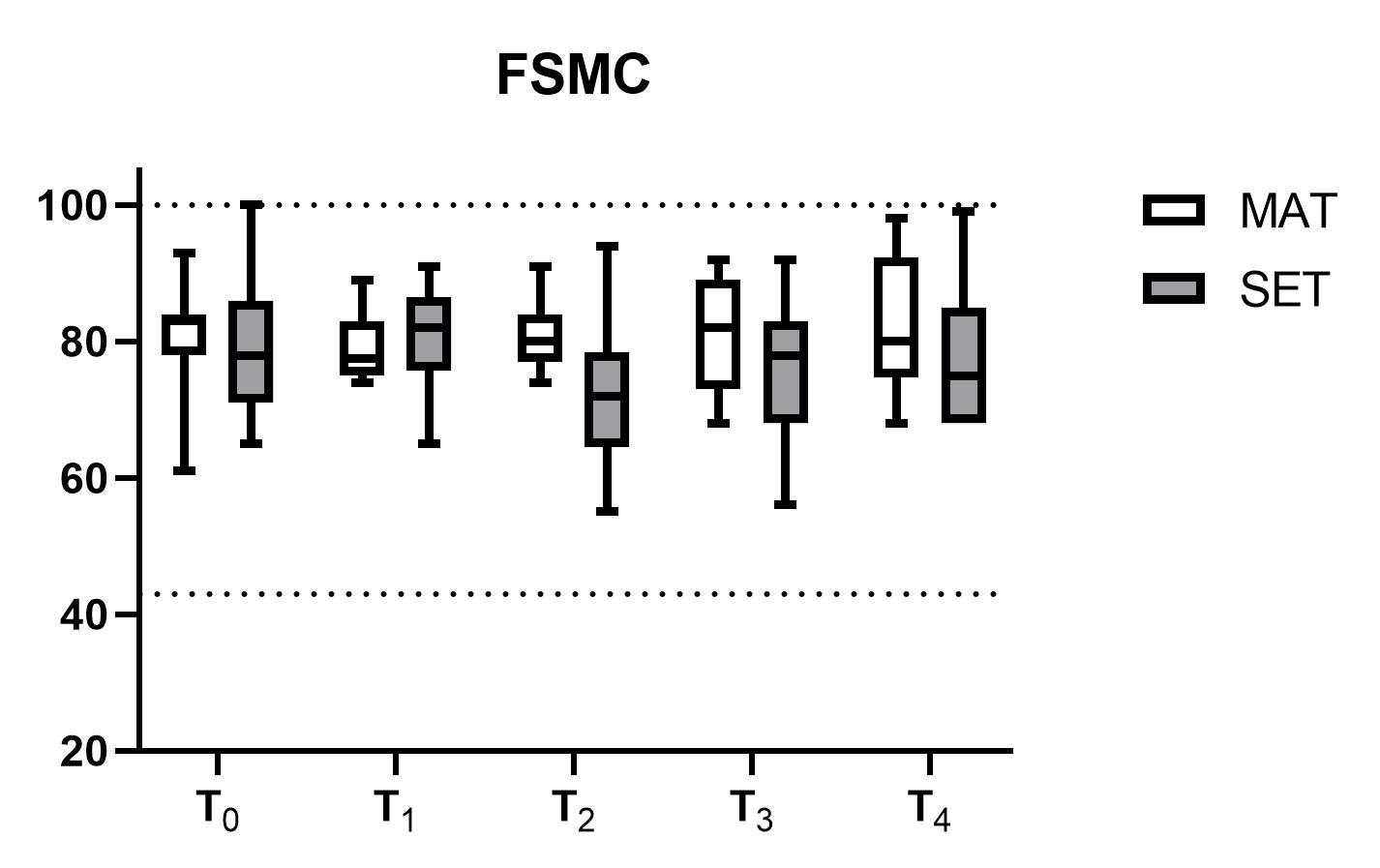
**

**Figure S1** FSMC total scores for both groups. Box plot: line=median, whiskers=min-max. Upper dotted line=maximum FSMC total score (=100); lower dotted line=cut-off for fatigue (=43); FSMC=Fatigue Scale for Motor and Cognitive Functions; T_0_=admission; T_1_=discharge; T_2_=1 week post-discharge; T_3_=4 weeks post-discharge; T_4_=12 weeks post-discharge.

**References**

1. Valet M, Stoquart G, de Broglie C, Francaux M, Lejeune T. Simplified indices of exercise tolerance in patients with multiple sclerosis and healthy subjects: A case-control study. Scand J Med Sci Sports. 2020;30(10):1908-17.

2. Schlagheck ML, Bansi J, Wenzel C, Kuzdas-Sallaberger M, Kiesl D, Gonzenbach R, et al. Complexity and pitfalls in maximal exercise testing for persons with multiple sclerosis. Eur J Neurol. 2023;30(9):2726-35.

3. Borg G. Anstrengungsempfinden und körperliche Aktivität. Dtsch Arztebl International. 2004;101(15):A-1016.

4. Foster C, Florhaug JA, Franklin J, Gottschall L, Hrovatin LA, Parker S, et al. A new approach to monitoring exercise training. J Strength Cond Res. 2001;15(1):109-15.

5. Langeskov-Christensen M, Langeskov-Christensen D, Overgaard K, Moller AB, Dalgas U. Validity and reliability of VO(2)-max measurements in persons with multiple sclerosis. J Neurol Sci. 2014;342(1-2):79-87.
